# The effect of a training webinar on decreasing inter-observer variability in making a radiologic diagnosis of bronchiectasis

**DOI:** 10.1186/s12880-022-00878-3

**Published:** 2022-08-24

**Authors:** O’Neil Green, Alexander Knee, Angelica Patino, Lucy Modahl, Sybille Liautaud

**Affiliations:** 1 UMASS-Baystate, 759 Chestnut St, Springfield, MA 01144 USA; 2 Department of Healthcare Delivery and Population Science, Baystate Medical Center, 759 Chestnut St, Springfield, MA 01144 USA; 3 Department of Radiology, UMASS-Baystate, 759 Chestnut St, Springfield, MA 01144 USA; 4 Department of Pulmonary and Critical Care Medicine, UMASS-Baystate, 759 Chestnut St, Springfield, MA 01144 USA

**Keywords:** Bronchiectasis, Web-based training, CT chest, Interpretation, Interrater reliability

## Abstract

Non-cystic fibrosis bronchiectasis is a clinically important disease with an estimated 340,000–522,000 persons living with the disease and 70,000 being diagnosed annually. The radiographic diagnosis remains a pivotal part of recognizing the disease due to its protean clinical manifestations. As physicians are sensitized to this disease, a greater proportion of patients are being diagnosed with mild to moderate bronchiectasis. Despite the established use of CT chest as the main tool for making a radiologic diagnosis of bronchiectasis, the literature supporting the process of making that diagnosis is somewhat sparse. Concurrently, there has been an increased trend to have Web-based radiologic tutorials due to its convenience, the ability of the learner to set the pace of learning and the reduced cost compared to in-person learning. The COVID-19 pandemic has accelerated this trend. We wanted to look carefully at the effect of a Web-based training session on interrater reliability. Agreement was calculated as percentages and kappa and prevalence adjusted kappa calculated. We found that a single Web-based training session had little effect on the variability and accuracy of diagnosis of bronchiectasis. Larger studies are needed in this area with multiple training sessions.

## Introduction

Bronchiectasis is a clinically heterogenous disease first described by Laennec in the 1800’s with further elaboration by Reid in the 1950’s [1]. Among subtypes of bronchiectasis, the data shows that the incidence of non-cystic fibrosis bronchiectasis is increasing worldwide [2]. Bronchiectasis is increasingly being diagnosed by computed tomography (CT) of the chest. Older methods such as CXR are less sensitive, and while bronchogram with iodinated contrast is more sensitive, it is mildly invasive and not without complications. Naidich first described the CT signs of bronchiectasis in 1982; the primary features include an elevated bronchial-to-arterial ratio and smooth dilation of bronchi to the periphery [3]. Other radiographic findings are sub-lobar collapse caused by mucus plugging, and strings/clusters of cysts. Despite this, studies which have looked at interrater reliability have used a variety of radiologic markers to define bronchiectasis. Most used the broncho-arterial ratio, but at cut-points between 0.9 and 1. Other markers which have been inconsistently used include lack of tapering of the bronchial lumen for longer than 2 cm and visualization of a bronchus within 1 cm of the costal pleura. Standardization of the CT acquisition protocol has also not been defined. Interrater reliability varies significantly across studies, from high (intraclass correlation 0.93–0.97) to modest (kappa 0.64–0.65) [4–6] Of course, where disease is severe, interrater reliability is high [7]. Various scoring systems have been used for assessing the severity of bronchiectasis including the Bhalla, BRICS, Santamaria and Oikonomou [8–12]. These are non-binary systems aimed at assessing severity of bronchiectasis and not validated for simple presence/absence of disease.

Web-based training for radiologic diagnoses is well entrenched in the medical curriculum, most notably with breast and spinal imaging. In the setting of the COVID-19 pandemic there has been a call to accelerate this trend as a mitigating countermeasure [12, 13]. Some authors have discussed convenience, the ability of the learner to set the pace of learning and the reduced cost compared to in-person learning [14, 15]. Despite this, there is some evidence to suggest that training using a web-based application results in at best minimal improvement in improving interrater reliability, sensitivity, specificity, and accuracy [16, 17]. To date, no study has assessed the role of Web-based training sessions in improving the diagnostic accuracy of chest CT scans for bronchiectasis. As such, the goal of this current research was to ascertain whether Web-based training would be beneficial in improving interrater reliability in making a diagnosis of bronchiectasis amongst attending physicians evaluating CT scans using the criteria of: a broncho-arterial ratio of ≥ 1, a lack of tapering of the bronchi toward the periphery, and the presence of peri bronchial thickening ± the presence of subsegmental collapse/consolidation. This research was done as a preliminary exploratory analysis of data obtained from a study investigating the accuracy of the ICD-9 and ICD-10 coding system in identifying patients with NCFB in a cohort of patients who have had a chest CT for an indication of bronchiectasis. We hypothesized that a training session would improve consensus amongst the physicians reading CT scans.

## Methods

In the pre-training phase, a sample of 40 cases were randomly selected as a computer-generated list from a larger cohort of 1500 cases chosen for an indication of bronchiectasis, or where bronchiectasis was in the final impression. We excluded images where traction bronchiectasis was in the final impression, or if the indication was for cystic fibrosis. Cases included dedicated and non-dedicated chest studies such as: HRCT Chest, CTA chest, CT chest with contrast, CT chest without contrast as well as CT abdomen/pelvis where the basal lung cuts could be accessed. This study was embedded in a larger study assessing the accuracy of ICD coding of NCFB. As part of this larger project, we initially designed an exploratory study to determine the accuracy of the initial radiologic diagnosis. During this process, we observed that agreement between reviewers was poor, therefore we developed a web-based training designed to improve intra-observer agreement. As this was an exploratory study, no sample size estimation was done. We chose a sample size of 40 for reasons of feasibility and considered it sufficient to identify issues related to accuracy of the initial radiologic diagnosis.

The reviewers had access to the complete study of each anonymized case through the IntelePACS® system (Intelerad Medical Systems Inc., Quebec, CA) and evaluated for bronchiectasis by freely scrolling through the case. Cases were assessed for presence or absence of bronchiectasis by three blinded independent reviewers (two pulmonologists with special interest and expertise in bronchiectasis, and a chest radiologist). There was no communication between raters during the rating period. The reviewers were aware that their judgements would be compared with other raters. All reviewers had the same set of images. The reviewers had limited access to clinical records beyond demographics and indication for CT chest. The review was done from hospital workstations with display resolution 1920 × 1080 with dual screens. Standard desktop monitors were used with range 22–24″. The reviewers were mid-career physicians with an average of 23 years practice post their basic medical degree. Bronchiectasis was defined as a broncho-arterial ratio of ≥ 1, a lack of tapering of the bronchi toward the periphery and the presence of peri bronchial thickening ± the presence of subsegmental collapse/consolidation. Results were then submitted in a yes/ no format on Excel spreadsheets independently prior to training. All methods were carried out in accordance with relevant guidelines and regulations.

**Fig. 1:**
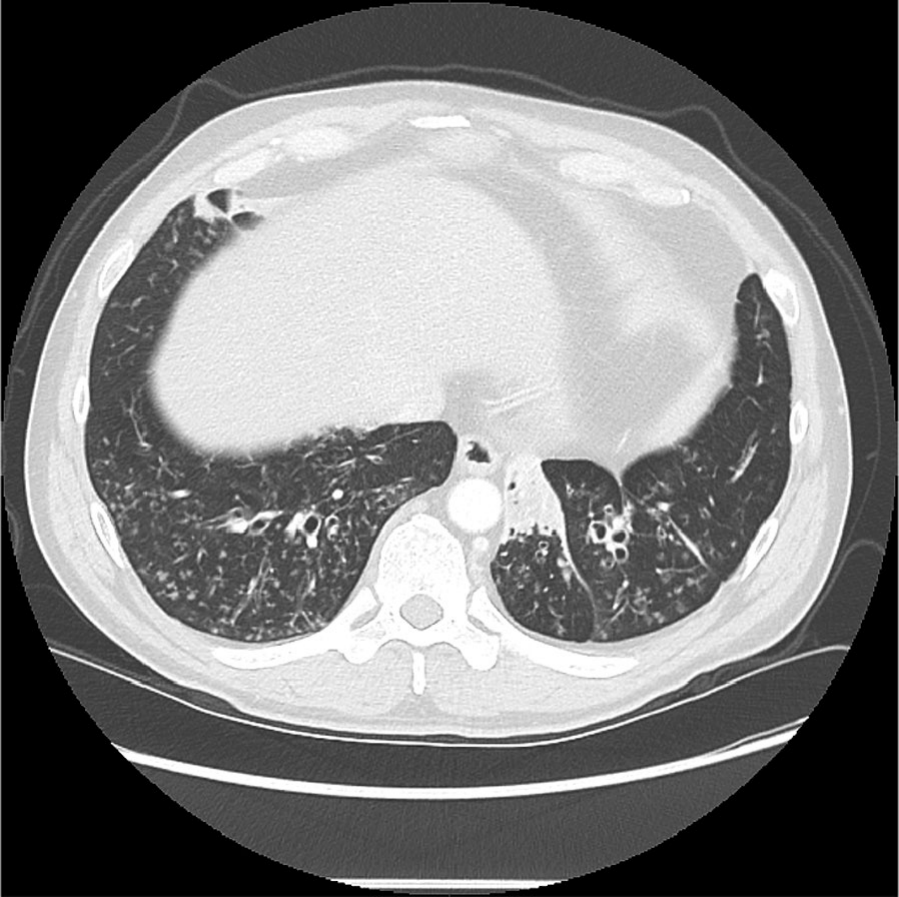
Representative CT cut of a subject with moderate bronchiectasis

Training of the reviewers consisted of a real time virtual session with a chest radiologist in which explanatory cases were shown and key points discussed. The session duration was approximately 40 min via WebEx with representative CT slices discussed, and the radiographic criteria re-iterated. Severe as well as mild/moderate cases of bronchiectasis were included. Figure 1 shows a representative CT slice of a subject with moderate bronchiectasis.

Four weeks after the Web-based training, a separate pool of 40 cases from the cohort were randomly selected and the same reviewers evaluated the CT images for the presence/absence of bronchiectasis using the same methods and criteria described above.

Inter-observer agreement for multiple raters was evaluated pre and post training using percent agreement, the kappa statistic and (due to low prevalence) prevalence adjusted kappa [18]. Estimates are presented with 95% confidence intervals using the command –kappaetc– [19]. Statistical analysis was conducted with Stata v16.1 (StataCorp, LP, College Station, TX).

## Results

**Fig. 2:**
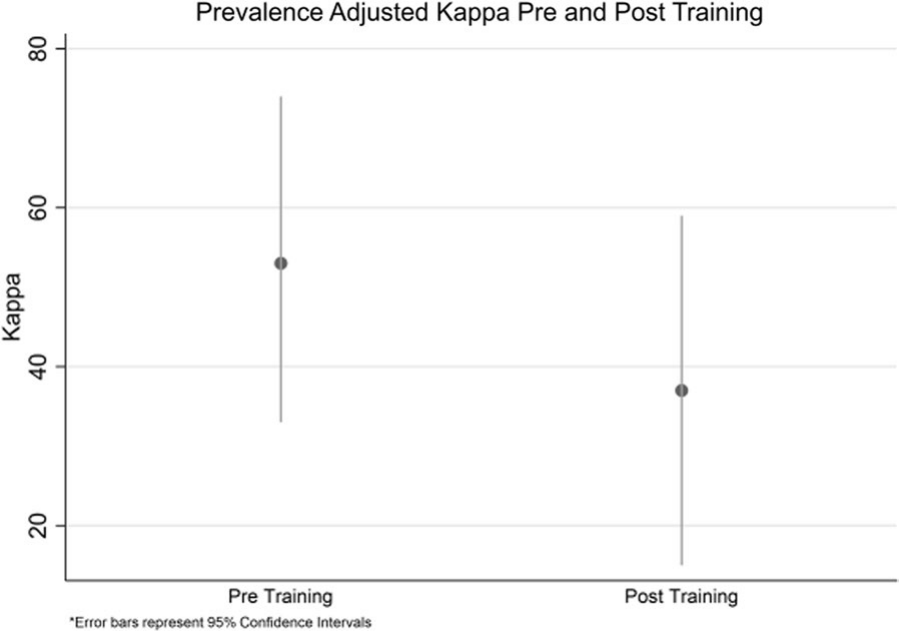
Comparison of interobserver variability in interpretation of bronchiectasis on CT before and after Web-based training

In our pre-training sample (n = 40), the percent agreement was 77% (95% CI 66–87%). Kappa was 0.22 (95% CI − 0.01 to 0.46) and prevalence adjusted kappa was 0.53 (95% CI 0.33–0.74). Of the 40 cases reviewed following training, two were excluded because of poor quality of the images, or the images could not be found in the PACS system. Percent agreement in the post training sample was 68% (95% CI 57–80%). Kappa was 0.25 (95% CI − 0.02 to 0.51) and prevalence adjusted kappa was 0.37 (95% CI 0.15–0.59) (Fig. 2).

We did not formally test whether the Kappa values were different before vs after the Webinar. However, based on our estimates presented in Fig. 1, the 95% confidence intervals overlap substantially suggesting we would have insufficient evidence to reject the null hypothesis of no difference.

### Pre-training

100% agreement (3/3) in 65% of the sample

- Pulm1/ Rads only agreement (15%)
- Pulm1/Pulm2 only agreement (12.5%)
- Pulm2/Rads only agreement (7.5%) (Table 1)

**Table 1:** Raw scores of individual raters before Web-based training

Pulm 1	Rads	Pulm 2	Frequency	Percentage
0	0	0	1	2.5
0	0	1	2	5
1	0	0	3	7.5
1	0	1	5	12.5
1	1	0	4	10
1	1	1	25	62.5

### Post-training

100% agreement (3/3) in 52.6% of the sample

- Pulm1/Rads only agreement (23.7%)
- Pulm1/Pulm2 only agreement (13.1%)
- Pulm2/Rads only agreement (10.5%) (Table 2)

**Table 2:** Raw scores of individual raters after Web-based training

Pulm 1	Rads	Pulm 2	Frequency	Percentage
0	0	0	4	10.53
0	1	0	2	5.26
0	1	1	2	5.26
1	0	0	2	5.26
1	0	1	3	7.89
1	1	0	9	23.68
1	1	1	16	42.11

## Discussion

Our data suggests that despite published criteria for making a diagnosis of bronchiectasis, there is significant variability in CT interpretation. This variability did not improve with a Web-based training session. In fact, variability increased instead of decreasing. However, the confidence intervals overlap substantially, and the sample size is quite small. A limitation of our findings is that this is a single center study using a relatively modest sample size. We employed Web-based training due to the constraints of the SARS-CoV-2 pandemic on in-person meetings at our institution. Web-based radiology training has been piloted in a variety of educational programs with mixed results [20–22]. There is some suggestion that Web-based learning impairs tacit learning which is an important means of skill transfer. The experience of the presenter in adapting training from live to Web-based training is an important determinant. Our presenter had extensive experience in Web-based training. Another issue may be the degree to which a short training session could modify interrater reliability in a group of physicians who already have significant experience in interpreting CT evidence of bronchiectasis. Post training, the consensus for presence of bronchiectasis decreased. This may have been due to increased sophistication gained in assessment of broncho-arterial ratio which was a key determinant of the radiologic diagnosis. There is still some subjectivity in deciding which bronchial/arterial pairing is assessed. There also is variation in deciding whether to use outer diameter vs inner diameter for bronchial measurements [23]. We used the inner diameter for assessment. Naidich did include wall thickening among other criteria in his diagnosis of bronchiectasis. These criteria tend to be somewhat subjective. We therefore reduced the criteria to those most frequently used in the literature and which were less subjective. The time that elapsed between training and interpretation may also have contributed to the variability due to recall bias. However, this may be reflective of real-world circumstances. More data is needed in this area.

We note that a significant number of our patients qualitatively had mild to moderate bronchiectasis, though we did not formally score for severity in this study. This may have contributed to the variability. Using the interpretation proposed by Zegers et al., the degree of reliability ranged from moderate to fair [24]. A series enriched for advanced cystic bronchiectasis would undoubtedly have less variability. With increasing awareness of the importance of non-cystic fibrosis bronchiectasis, many patients seen will have modest changes on CT chest despite significant symptoms related to their disease. In practice, both clinicians and radiologists have access to extensive clinical records as well as serial lung imaging studies which provide a wealth of data and will alter the interpretation of the interval CT chest image. A recent international consensus suggested a stepwise approach to diagnosis that includes clinical findings [23]. Clearly, radiologists have recognized formal training in diagnosing bronchiectasis on CT chest images. However, pulmonologists may have patients referred to them with chest complaints and CT images depicting bronchiectasis which were missed by the initial radiologist, and there is an expectation that pulmonologists should be able to recognize disease patterns on lung images.

Variability in recognition of the CT changes has implications for the timely recognition of the disease and follow-up. Though modest data is published on longitudinal follow-up of patients with moderate bronchiectasis and in identifying factors which lead to progression of disease, there clearly are sub-groups of patients who require close follow from early in their disease trajectory, such as patients with bronchiectasis as a co-diagnosis of nontuberculous mycobacteria infection [25]. Given that interrater reliability is so low, our findings may also suggest that there may be utility in a radiologic consensus opinion particularly in the setting of some subtle disease or questionable radiographic patterns which may be more challenging calls. Moreover, in dedicated chest clinics, whenever there is suspicion of bronchiectasis, a multidisciplinary approach may be best with more than one reviewer looking at the CT chest with a clear set of diagnostic guidelines in mind. The utility of consensus panels may offer unique opportunities evaluating the approach to reading CT scans for bronchiectasis in the future. Other methods have been proposed such as quantitative CT imaging [26, 27]. However, many patients with bronchiectasis live in resource-poor regions of the world and the technology remains imperfect, often limiting quantitative CT imaging to research settings. Therefore, skilled radiology assessment of bronchiectasis on CT chest remains the most common method of detection of disease and assessment of progression.

The variety of image acquisition methods may affect the interpretation and it is possible that if the images had standardized acquisition, i.e. multi-detector CT with contiguous 1-mm slices, we may have had increased interrater reliability. However, this study reflects real-world conditions as many patients will be diagnosed based on retrospective images acquired in a variety of CT formats.

## Conclusion

The effect of a Web-based training session on reducing interobserver variability and increasing accuracy in making a radiologic diagnosis of bronchiectasis appears limited. Despite this, the increasing availability of the Web, the convenience of Web-based learning, and the cost-effectiveness of this approach will contribute to an increase in its application. More studies are needed in this area. The radiologic diagnosis of bronchiectasis is challenging particularly where the disease is mild to moderate. Multiple CT reviewers may improve the sensitivity of the diagnosis especially in this subset. In clinics dedicated to the management of bronchiectasis, a multidisciplinary approach to diagnosis may result in improved recognition of these patients.

## Data Availability

The datasets generated and/or analyzed during the current study are not publicly available due the fact that they are part of the University of Massachusetts Health System REDCap database which does not allow this option but are available from the corresponding author on reasonable request.

## References

[1] Hayward J, Reid L (1952). The cartilage of the intrapulmonary bronchi in normal lungs, in bronchiectasis, and in massive collapse. Thorax.

[2] Seitz AE, Olivier KN, Adjemian J (2012). Trends in bronchiectasis among Medicare beneficiaries in the United States, 2000 to 2007. Chest.

[3] Naidich DP, McCauley DI, Khouri NF (1982). Computed tomography of bronchiectasis. J Comput Assist Tomogr.

[4] Wu J, Bracken J, Lam A (2021). Refining diagnostic criteria for pediatric bronchiectasis using low-dose CT scan. Resp Med.

[5] Dodd J, Souza C, Muller N (2006). Conventional high-resolution CT versus helical high-resolution MDCT in the detection of bronchiectasis. Am J Roentgenol.

[6] Tiddens H, Meerburg J, van der Eerden M, Ciet P (2020). The radiological diagnosis of bronchiectasis: what's in a name?. ERJ.

[7] Edwards EA, Metcalfe R, Milne DG (2003). Retrospective review of children presenting with non-cystic fibrosis bronchiectasis: HRCT features and clinical relationships. Pediatr Pulmonol.

[8] Bhalla M, Turcios N, Aponte V (1991). Cystic fibrosis: scoring system with thin-section CT. Radiology.

[9] Pallavi B, Chalmers J, Goeminne P (2018). A multicenter study score for use in idiopathic and postinfective bronchiectasis. Chest.

[10] Santamaria F, Grillo G, Guidi G (1998). Cystic fibrosis: when should high-resolution computed tomography of the chest be obtained?. Pediatrics.

[11] Oikonomou A, Manavis J, Karagianni,  (2002). Loss of FEV1 in cystic fibrosis: correlation with HRCT features. Eur Radiol.

[12] Weinberger E, Iakobovits R, Halsted M (2002). My PACS.net: a web-based teaching file authoring tool. Am J Roentgenol.

[13] Virarkar M, Jensen C, Javadi S (2020). Radiology education amid COVID-19 pandemic and possible solutions. J Comput Assist Tomogr.

[14] Uadhyay N, Wadkin JCR (2021). Can training in diagnostic radiology be moved online during the COVID-19 pandemic? UK trainee perceptions of the Radiology-Integrated Training Initiative e-learning platform. Clin Radiol.

[15] Selvaggi S, Sicignano G, Vollono E (2008). E-learning tools for education: regulator aspects, current applications in radiology and future prospects. Radiol Med.

[16] Buijze G, Guitton TG, Niek van Dijk C (2012). Training improves interobserver reliability for the diagnosis of scaphoid fracture displacement. Clin Orthop Relat Res.

[17] Peng JM, Qian CY, Yu XY (2017). Does training improve diagnostic accuracy and inter-rater agreement in applying the Berlin radiographic definition of acute respiratory distress syndrome? A multicenter prospective study. Crit Care.

[18] Gwet KL (2014). Handbook of inter-rater reliability: the definitive guide to measuring the extent of agreement among raters.

[19] Klein D. KAPPATEC: Stata module to evaluate interrater agreement. Statistical Software Components S458283, Boston College Department of Economics, revised 06 Jan 2019; 2016.

[20] Grunewald M, Heckemann RA, Gebhard H (2003). COMPARE radiology: creating an interactive Web-based training program for radiology with multimedia authoring software. Acad Radiol.

[21] Wade S, Moscova M, Tedla N (2020). Adaptive tutorials versus web-based resources in radiology: a mixed methods analysis in junior doctors of efficacy and engagement. BMC Med Educ.

[22] Ayesa S, Katelaris A, Brennan P, Grieve S (2021). Medical imaging education opportunities for junior doctors and non-radiologist clinicians: a review. J Med Imaging Radiat Oncol.

[23] Aliberti S, Goeminne P, O’Donnell A (2021). Criteria and definitions for the radiological and clinical diagnosis of bronchiectasis in adults for use in clinical trials: international consensus recommendations. Lancet Respir Med.

[24] Zegers M, de Bruijne MC, Wagner C (2010). The inter-rater agreement of retrospective assessments of adverse events does not improved with two reviewers per patient record. J Clin Epidemiol.

[25] Kitada S, Uenami T, Yoshimura K (2012). Long-term radiographic outcome of nodular bronchiectatic Mycobacterium avium complex pulmonary disease. Int J Tuberc Lung Dis.

[26] Gaik CO, Pek LK, Chan-Yeung M (2002). High-resolution CT quantification of bronchiectasis: clinical and functional correlation. Radiology.

[27] Ledda RE, Balbi M, Milone F (2021). Imaging in non-cystic fibrosis bronchiectasis and current limitations. BJR Open.

